# Epidemiology of unplanned intensive care admissions through inhospital referrals at a tertiary referral centre university hospital

**DOI:** 10.1186/cc13274

**Published:** 2014-03-17

**Authors:** J Rigby, N Bradshaw, B Carr, R Matsa

**Affiliations:** 1 University Hospital of North Staffordshire, Stoke-on-Trent, UK

## Introduction

The provision of intensive care has lagged behind demand [1]. Intensive care services in the UK have significant resource restriction. Although rationing of beds has been a priority, equally important is to establish patient safety and also identify strategies to prevent admissions [2], which echoes the importance of early recognition of deteriorating patients and prompt intervention. This study evaluates the epidemiology of unplanned admissions to the ICU in order to identify the associative trends that led to the admission and hence to mitigate the processes of interventional strategies.

## Methods

A prospective observational evaluation of ICU unplanned admission through in-hospital ward referral over a 3-month duration. Anonymised data collection included baseline demographics, timing and grade of referral, Modified Early Warning Score (MEWS) at time of referral, clinical reason for referral, senior review prior to referral, MEWS trend 24 hours prior to referral, if appropriate basic intervention commenced, time delay between referral and assessment by the ICU team, organ failure score, length of stay (LOS) and survival status.

## Results

So far 22 patients have been enrolled, of which 60% of the unplanned admissions were 'out of hours', 60% of admissions were from medical wards and 33% from surgical wards. Pneumonia was the main reason for referral and admissions were due to respiratory failure requiring advanced ventilator care. Twenty per cent of the patients did not have senior medical review for at least 4 hours prior to the ICU referral. Basic interventions such as antibiotics and intravenous fluids were not started in a few patients (13%) prior to admission. The mean ICU review time from referral was 70 minutes and the mean MEWS at the time of referral was 8.

## Conclusion

The study is due to be completed by mid-January 2014. With the limitation of incompleteness, the findings so far have alluded that there is a necessitation for change and education for early identification and intervention in deteriorating patients. The completed study with outcomes (that is, survival rates and ICU LOS) will help us identify whether delay or deficiency in the early management had a causal relationship.
